# Methodological synthesis of Bayesian phylodynamics, HIV-TRACE, and GEE: HIV-1 transmission epidemiology in a racially/ethnically diverse Southern U.S. context

**DOI:** 10.1038/s41598-021-82673-8

**Published:** 2021-02-08

**Authors:** Kayo Fujimoto, Justin Bahl, Joel O. Wertheim, Natascha Del Vecchio, Joseph T. Hicks, Lambodhar Damodaran, Camden J. Hallmark, Richa Lavingia, Ricardo Mora, Michelle Carr, Biru Yang, John A. Schneider, Lu-Yu Hwang, Marlene McNeese

**Affiliations:** 1 Department of Health Promotion and Behavioral Sciences, The University of Texas Health Science Center at Houston, 7000 Fannin Street, UCT 2514, Houston, TX 77030 USA; 2 Department of Infectious Diseases, University of Georgia, Athens, GA USA; 3 Department of Medicine, University of California San Diego, La Jolla, CA USA; 4 Department of Biostatistics and Data Science, The University of Texas Health Science Center at Houston, Houston, TX USA; 5 Institute of Bioinformatics, University of Georgia, Athens, GA USA; 6 Division of Disease Prevention and Control, Houston Health Department, Houston, TX USA; 7 Department of Medicine, University of Chicago, Chicago, IL USA; 8 Department of Epidemiology, Human Genetics, and Environmental Science, The University of Texas Health Science Center at Houston, Houston, TX USA

**Keywords:** Bioinformatics, HIV infections, Population genetics, Risk factors, Statistics

## Abstract

This study introduces an innovative methodological approach to identify potential drivers of structuring HIV-1 transmission clustering patterns between different subpopulations in the culturally and racially/ethnically diverse context of Houston, TX, the largest city in the Southern United States. Using 6332 HIV-1 *pol* sequences from persons newly diagnosed with HIV during the period 2010–2018, we reconstructed HIV-1 transmission clusters, using the HIV-TRAnsmission Cluster Engine (HIV-TRACE); inferred demographic and risk parameters on HIV-1 transmission dynamics by jointly estimating viral transmission rates across racial/ethnic, age, and transmission risk groups; and modeled the degree of network connectivity by using generalized estimating equations (GEE). Our results indicate that Hispanics/Latinos are most vulnerable to the structure of transmission clusters and serve as a bridge population, acting as recipients of transmissions from Whites (3.0 state changes/year) and from Blacks (2.6 state changes/year) as well as sources of transmissions to Whites (1.8 state changes/year) and to Blacks (1.2 state changes/year). There were high rates of transmission and high network connectivity between younger and older Hispanics/Latinos as well as between younger and older Blacks. Prevention and intervention efforts are needed for transmission clusters that involve younger racial/ethnic minorities, in particular Hispanic/Latino youth, to reduce onward transmission of HIV in Houston.

## Introduction

Although the rate of diagnosis of human immunodeficiency virus (HIV) in the United States (U.S.) decreased between 2011 and 2015, certain subpopulations bear an excess burden of new HIV diagnoses^1^. The highest HIV diagnosis rates were reported primarily for the subgroups of younger age, racial/ethnic minorities, men who have sex with men (MSM), and those who reside in the Southern region of the U.S.^1^. HIV-1 phylogenetic analysis has been increasingly used as a powerful methodological tool to reconstruct the HIV-1 transmission network by inferring putative HIV-1 transmission clusters formed by highly similar HIV strains. A number of HIV-1 molecular epidemiological studies have identified transmission clusters by assessing the viral genetic distance between these individual sequences^2–7^. Small genetic distances imply that individuals may have been infected from a common source in an overlapping time period. The shared common ancestry of a phylogenetic transmission cluster (or network) implies that members in that cluster share epidemiological characteristics of transmission. When combined with individuals’ demographic and transmission risk attributes, putative transmission clusters formed by highly similar HIV strains enable us to make inferences about clusters of epidemiologically connected individuals.

A number of HIV-1 molecular epidemiological studies have identified subpopulations in transmission networks that were characterized in terms of cluster membership, cluster size, and the degree of connectivity within a cluster. For instance, younger individuals, males, MSM, and heterosexuals tend to be members of a cluster^6,8–10^, while Black/African American (hereafter Black) individuals tend to cluster at lower frequencies than do White and Hispanic/Latino (hereafter Hispanic) individuals across the U.S.^5,11–13^. Within a transmission cluster, the degree of connectivity to other persons who are living with HIV increases for younger individuals, men, Blacks, and MSM^4^. Across three major U.S. metropolitan areas, Hispanic individuals cluster at high frequencies, whereas Black individuals cluster at the lowest frequencies and are more assortative than other race/ethnicity groups^11^.

Previous studies have identified and characterized emerging local transmission clusters and associated them with certain epidemiological profiles of co-clustered individuals in HIV-1 transmission networks. Little is known, however, about the drivers of HIV-1 transmission patterns between different vulnerable subpopulations who may fuel local HIV-1 sub-epidemics. As HIV-1 phylogenetic analysis is limited in its capability to establish directionality of transmission between individual members within these clusters^14^, it can provide only limited information in the absence of additional epidemiological data. As a consequence, there is scant knowledge of the epidemiological profiles of subpopulations who may, in fact, play a central role in the structuring of transmission clusters by acting as a recipient or source of HIV-1 infection.

In the present study, our primary goal was to identify potential drivers of structuring HIV-1 transmission clustering patterns between different subpopulations who act as sources and recipients of HIV-1 infection in the culturally and racially/ethnically diverse context of Houston/Harris County, TX. Our study took a hybrid heuristic and Bayesian approach^15^ and introduced an innovative methodology by synthesizing the HIV-TRAnsmission Cluster Engine (HIV-TRACE), Bayesian phylodynamics, and generalized estimating equations^16^ (GEE). Our approach enabled us to reconstruct HIV-1 transmission clusters; infer demographic and risk parameters of HIV-1 transmission dynamics by jointly estimating viral transmission rates across racial/ethnic, age, and transmission risk groups; and assess the effects of network mixing patterns based on racial/ethnic and age characteristics of the index person and partners on the degree of network connectivity. Our study is expected to inform the epidemiological profile of subpopulations with a disproportionate vulnerability and whose membership in clusters could be prioritized for urgent intervention efforts to eliminate the HIV epidemic in Houston.

## Methods

### Study setting and data

HIV molecular sequence data were reported from drug-resistance genotyping for people newly diagnosed with HIV while residing in Houston/Harris County or currently (as of January 1, 2019) residing in Houston/Harris County. Data were collected by the Houston Health Department as part of the routine HIV Surveillance Program. These samples were then stored in the Enhanced HIV/AIDS Reporting System. A total of 32,739 individuals older than 13 years and with an adult transmission risk were diagnosed in or currently residing in Houston/Harris County and entered into the surveillance system on or before December 31, 2018. Reporting of HIV genotypic testing has been required by law (Tex. Adm. Code Chapter 97, Subchapter F, §97.133) since January 1, 2010. Therefore, due to incompleteness of data prior to 2010, only individuals diagnosed in 2010 or later were included for analysis (*N* = 12,344).

The selection criteria for genotypes were: (1) had a reverse transcriptase (RT) or protease (PR)/RT HIV-1 *pol* sequence that was not ≤ 0.015 substitutions/site from the HXB2 reference sequence, and (2) had the month and year of sample date available. Further, PR and RT genotype were combined if they were reported at the same time but as separate sequences, and only the first PR/RT or RT genotype was used for constructing the network. Among 12,344 persons who were living with HIV diagnosed in or after 2010, 6516 (52.8%) had at least one HIV-1 sequence. Of the 6516 people with a sequence, 6332 had HIV-1 *pol* sequences that were used in the analysis.

This study was deemed to be exempt from institutional review by the Committee for the Protection of Human Subjects at the University of Texas Health Science Center at Houston because it was a retrospective analysis of surveillance data for the purposes of program evaluation. The data were analyzed in accordance with a Memorandum of Understanding for data sharing between the Houston Health Department and each institution. All methods were carried out in accordance with relevant guidelines and regulations.

The data that support the findings of this study are available from the corresponding author upon reasonable request. The data are not publicly available due to concerns regarding participant confidentiality.

### Measures

We used the following study variables: age (Younger coded as individuals born after 1990, Older coded as individuals born in or before 1990); sex assigned at birth (Male, Female); race/ethnicity (Hispanics refers to Hispanics/Latinos of all races, Blacks refers to non-Hispanic Blacks, Whites refers to non-Hispanic Whites, Asians refers to non-Hispanic Asians, Others refers to non-Hispanic other racial categories, including American Indians and Alaskan Natives, Multi-race refers to non-Hispanic multi-race categories); HIV transmission risk (MSM refers to cisgender men who reported MSM, Cisgender men refers to other cisgender men who did not report injection drug use (IDU) or MSM, Cisgender women refers to cisgender women who did not report IDU, Transgender women refers to transgender women who did not report IDU, and PWID (people who inject drugs) refers to anyone who reported injection drug use, including MSM); first viral-load test result after HIV diagnosis (< 10,000, 10,000–100,000, > 100,000 copies/ml); and first CD4^+^ T-cell count test result after HIV diagnosis (< 50, 50–200, 201–350, > 350 cells/mm^3^). We defined age groups using a birth-year cutoff rather than age cohorts, as our data came from participants who were diagnosed over a range of years and aged through the life of our clusters.

### Phylogenetic reconstruction and analysis

**Figure 1:**
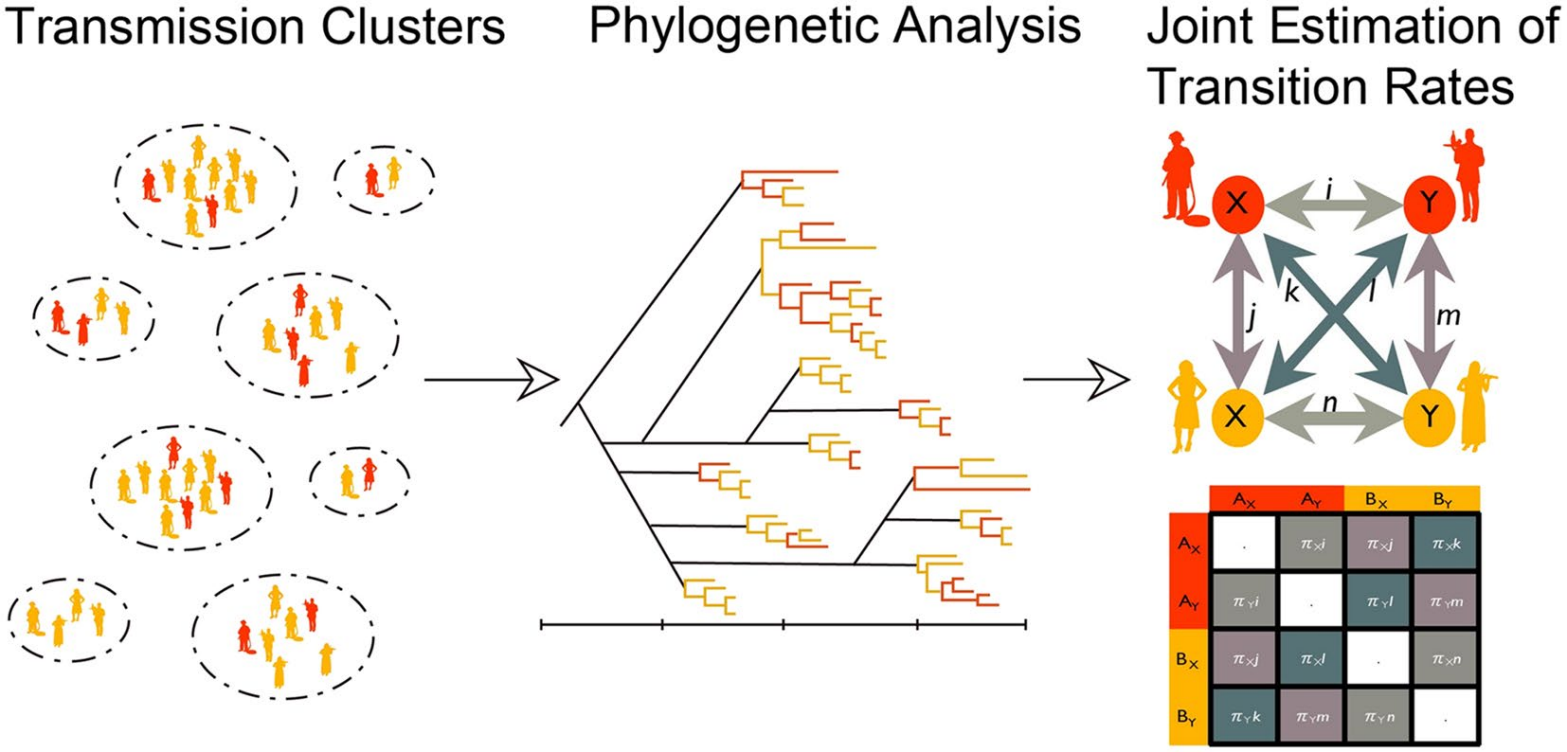
Phylogenetic analytical design. The three-step process of estimating viral transmission between epidemiological characteristics that structure HIV transmission clusters within Houston/Harris County is summarized. In the first step, transmission clusters were identified. In the second step, a phylogenetic analysis was conducted to estimate the time of the most recent common ancestor for each cluster. In the third step, a joint estimation of transition rates of a single viral transition matrix for all clusters was conducted.

Testing hypotheses to identify underlying characteristics of HIV transmission clusters is challenging because most clusters contain few individuals, and, therefore, unbiased statistical inference is often not possible. To overcome this, we used a joint estimation procedure, whereby we investigated the relationships between transmission cluster characteristics and three epidemiological characteristics of race/ethnicity, age, and transmission risk to identify how these epidemiological characteristics may structure transmission clusters within Houston/Harris County. Our phylogenetic analysis design followed a three-step process: (1) identification of transmission clusters, (2) estimation of the time of the most recent common ancestor for each cluster, and (3) joint estimation of a single viral transition matrix for all clusters. For Steps 2 and 3, our phylogenetic analysis was based on the use of clusters with five or more members and exclusion of homogeneous clusters for computational tractability and the ability to infer rates that were meaningful and realistic across all identified clusters. Figure 1 provides a visual summary of our analytical design.

In Step 1, we constructed a genetic transmission network, using HIV-TRACE^17,18^. All sequences were aligned to an HXB2 reference sequence (positions 2253–3749). To identify genetically linked pairs, we used a pairwise genetic distances threshold of 0.015 substitutions/site and an ambiguity fraction of 1.5%.

In Step 2, we estimated a preliminary maximum likelihood phylogenetic tree, using a general time-reversible model with gamma distributed rate variation among sites (GTR Gamma), using RAxML v8.2.4^19^. The “clocklikeness” of the tree was determined using TempEst v.1.5.3 with a root-to-tip linear regression^20^. Then, we conducted statistical phylodynamic analysis using BEAST v 1.10.1^21^. All sequences were aligned to generate a rooted, time-measured phylogeny, inferred using a relaxed molecular clock model to estimate the time of the most recent common ancestor of each cluster. We used the GTR 4 substitution model that accounts for the proportion of invariant sites that does not undergo evolutionary change.

In Step 3, to assess the importance of epidemiological parameters in HIV transmission, we performed an ancestral state reconstruction^22^ of racial/ethnic and transmission risk characteristics using Bayesian Stochastic Search Variable Selection (BSSVS). For phylodynamic analysis, we used clusters constructed using a more conservative genetic distance threshold of 0.005 substitutions/site. This lower threshold identifies more recent potential transmission partners^23^ with fewer intermediate partners. Intermediate partners can confound estimates of frequencies of transmission among risk groups^24^. We estimated a single discrete state transition rate matrix jointly across all clusters independently instead of one matrix for a tree that contains all clusters under the assumption that underlying characteristics are shared by all clusters in Houston/Harris County^25^.

We jointly estimated a single phylodynamic discrete trait model to all clusters for each trait of race/ethnicity and transmission risk. We used this joint estimation approach because epidemiological characteristics of a population can determine the structure of outbreaks. Individual clusters, however, are linked on a phylogenetic tree by long branches. These branches represent unobserved members of an outbreak, viral migration, or a biological change that triggered the outbreak. Phylodynamic approaches to hypothesis testing, including ancestral state reconstruction of tip-associated discrete traits, assume that the rate of change is constant throughout the branches of the phylogenetic tree. Although this assumption is reasonable for individual clusters, it is unlikely to be valid for the entire tree. Therefore, a jointly fitted single discrete model should capture the signal of the population’s social structure that may determine patterns of HIV transmission.

The tip-associated traits that we analyzed included race/ethnicity, race/ethnicity plus year of birth (born in or before 1990 and after 1990), and transmission risk. We used an asymmetric continuous-time Markov chain model^26^ for discrete state reconstructions and performed three independent Markov chain Monte Carlo runs with a chain length of 10 million states, logging every 1000 for each model. We applied ancestral state reconstruction of a racial/ethnicity discrete state model to an empirical set of 1500 phylogenetic trees for each cluster.

Given the large number of states, BSSVS was employed to search parameter space and identify those parameters with significantly non-zero transition rates^22,27^. BSSVS explores and efficiently reduces the state space by employing a binary indicator (I)^22,27^. From the BSSVS results, we applied a Bayes Factor (BF) test to assess the support for individual transitions between discrete states. We removed a burn-in of 10% (100,000) and computed BF values using SpreaD3^27^. We used the following scheme to assess the level of support: No support: BF < 3; substantial support: BF = 3–10; strong support: BF = 11–30; very strong support: BF = 31–100; decisive support: BF > 100. More details for Steps 2 and 3 are provided in the online supplemental materials.

### Population averaged negative binomial models for connectivity

We conducted a supplementary statistical analysis to account for homogeneous clusters with regard to epidemiological characteristics in our phylogenetic analysis, as well as to control for clinical factors. We estimated GEE for our data clustered on identified phylogenetic clusters with five or more members. We conducted negative binomial regression analysis to model the connectivity level (i.e., network degree) as a function of the number of phylogenetically connected partners with matched or mismatched attributes of race/ethnicity (Blacks, Hispanics, Whites, and Asians/Others) and age (Younger and Older) with the index person. In this analysis, we created eight assortative mixing terms by computing the number of partners who are matched with an index on these subcategories as well as 42 disassortative mixing terms by computing the number of partners who are mismatched. Our model controlled for clinical factors (log-transformed viral load values, CD4^+^ T-cell count level), diagnosis year (2010–2018), and other sociodemographic and risk variables. We used an exchangeable correlation structure with empirical variance estimates to address potential mis-specifications on the correlation structure. We used the *xtnbreg* command implemented in Stata v16.0 (College Station, TX).

## Results

### Descriptive statistics

A majority of the 6332 individuals in our sample were older (born in or before 1990) (81%), males (80%), of a racial/ethnic minority (47% Blacks and 36% Hispanics), and MSM (54%). In addition, 42% had an initial viral load after HIV diagnosis greater than 10,000 copies/ml, and 88% had a CD4^+^ T-cell count of > 200. The detailed descriptive statistics for sample characteristics are presented in Supplemental Table 1S in the supplemental materials.

### Phylogenetic analysis

#### Cluster identification

**Figure 2:**
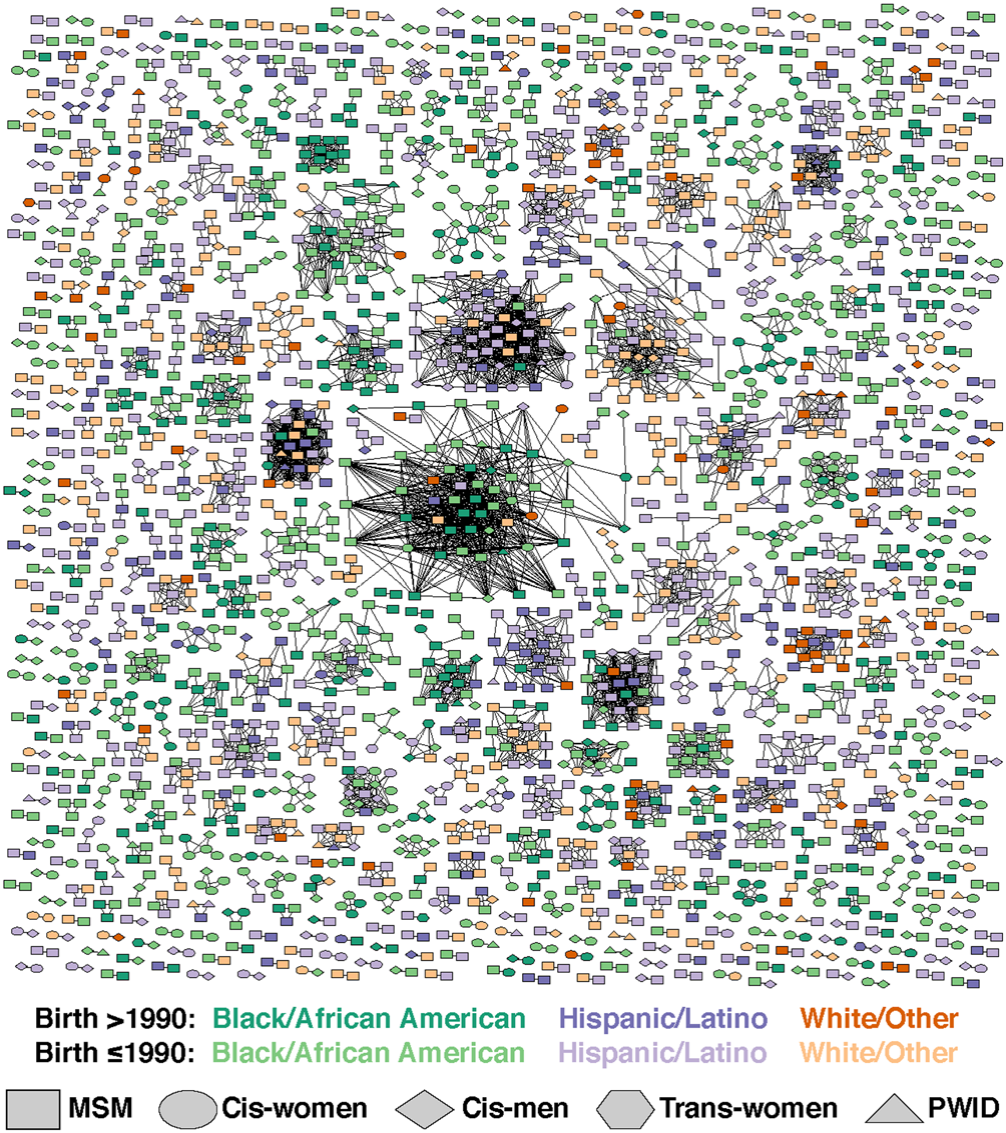
Visualization of HIV-1 transmission network in Houston/Harris County (2010–2018). MSM refers to cisgender men who report being MSM, cis-women refers to cisgender women who did not report injection drug use, trans-women refers to transgender women who did not report injection drug use, cis-men refers to cisgender men who did not report injection drug use or MSM, and PWID refers to anyone who reported injection drug use, including MSM. Hispanic/Latino refers to Hispanic/Latino of all races; Black/African American refers to non-Hispanic Black/African American; and White/Other refers to non-Hispanic Whites, non-Hispanic Asians, and non-Hispanic other racial categories, including American Indians, Alaskan Natives, and multi-race categories.

We identified 2675 (42%) samples with a cluster size of two or more out of 6332 samples, with a mean cluster size of 12 (SD = 14.2, min = 2, max = 58). Among these 2675 samples, 1529 (57%) belonged to 143 unique clusters, with five or more members with a mean cluster size of 18.5 (SE = 15.7, min = 5, max = 58). The categories of younger individuals (38%), males (27%), Whites (27%), MSM (31%), viral load of 10,000–100,000 (26%), and CD4^+^ T-cell count of > 350 (28%) showed a higher percentage of membership within a cluster that comprised ≥ 5 members (Supplemental Table 1S). An HIV-1 putative transmission network by racial/ethnic groups and risk categories is presented in Fig. 2.

There were three large clusters, two of which were composed mainly of heterosexual males with minority racial/ethnic groups.

#### Comparative phylogenetic analysis

The meta-data of epidemiological profile variables associated with each cluster allowed us to estimate the transmission network that connects these different racial/ethnic groups. Out of 143 identified clusters with five or more members who were identified by our TRACE analysis, we identified 34 racially/ethnically non-homogeneous transmission clusters and 27 non-homogeneous clusters with respect to transmission risk categories, using a more conservative genetic distance threshold of 0.005 substitutions/site. It is important to note that, even though the majority of transmission is within racial/ethnic groups, the contact rates between groups is an important characteristic that can determine viral spread. We therefore excluded these homogeneous clusters from our analysis.

**Figure 3:**
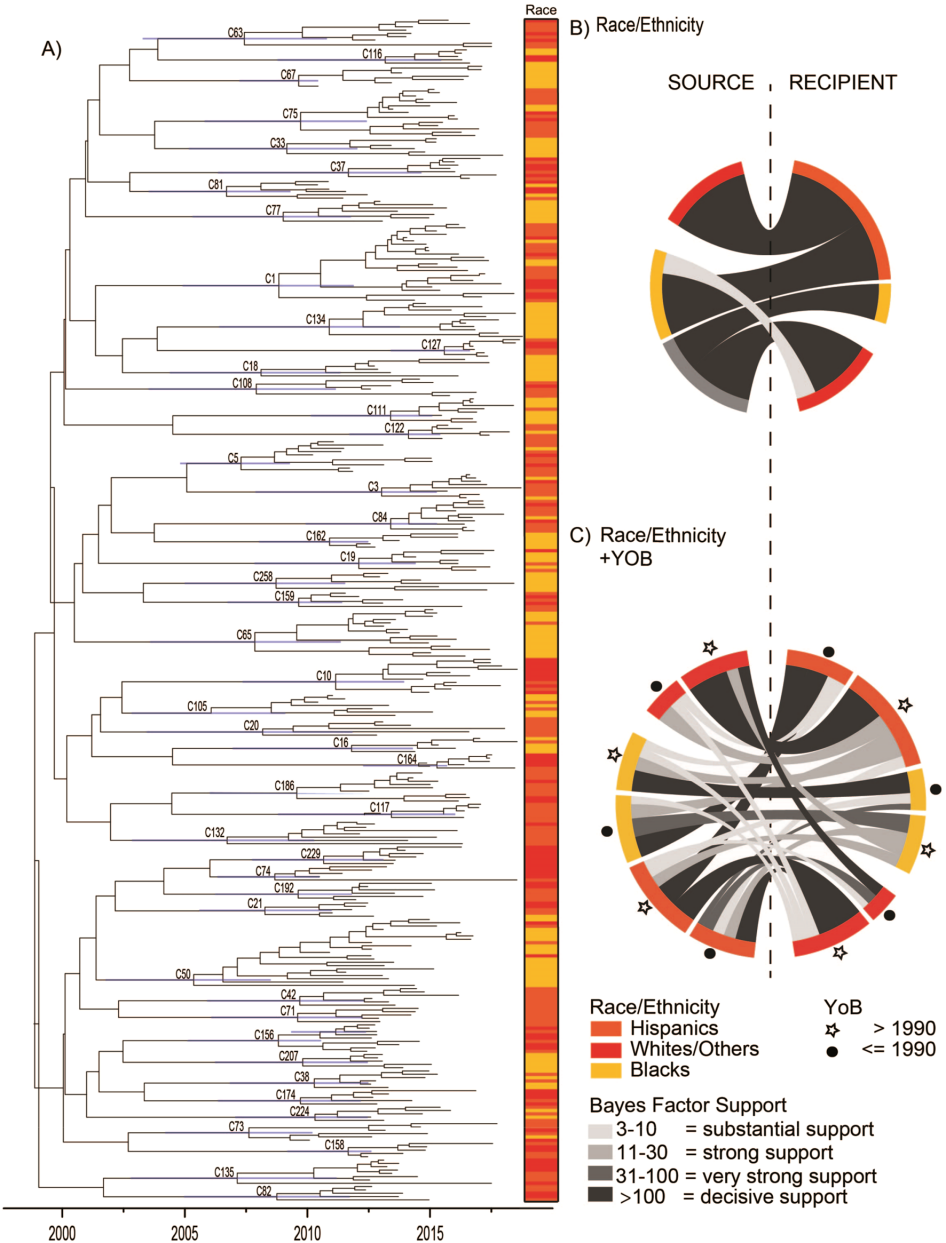
(**A**) BEAST phylogeny of all clusters; (**B**) chord diagram for viral transmission between racial/ethnic groups (Hispanics, Blacks, Whites/Others); (**C**) chord diagram for viral transmission between racial/ethnic groups plus age group (born in or before 1990, born after 1990). (**A**) Shows a maximum clade credibility phylogenetic tree with all clusters that contain five or more cluster members. Tips are labeled with the race/ethnicity associated with the taxa at that tip. In (**B**) and (**C**), chord thickness indicates the magnitude of the transition rate, and color indicates Bayes Factor support. Only supported transition rates from discrete trait analysis are displayed. *YoB* Year of Birth.

Members of these clusters were diagnosed between 2010 through the end of 2018. Phylogenetic analysis of the combined groups was conducted to determine the time of the most recent common ancestor for each cluster (Fig. 3A). Clusters circulated for 3.45 to 13.56 years, with larger clusters as circulating for longer periods. We estimated transmission between racial/ethnic groups, using an ancestral state reconstruction method. We assume that the underlying demographic characteristics are shared by all clusters, and, therefore, we jointly estimated a single transmission network shared among all clusters analyzed.

#### A single discrete race/ethnicity model

Figure 3B shows a chord diagram that indicates viral transmission between different groups of races/ethnicities. We used an asymmetric model to allow for the directionality of transmission to be estimated. We observed decisively significant transitions (BF > 100) from Whites to Hispanics (2.99 state changes/year), from Blacks to Hispanics (2.55 state changes/year), and from Hispanics to Whites (1.84 state changes/year) (Fig. 3B, Supplemental Tables 3S). Further, Hispanics had decisively supported transitions (BF > 100) to Asians (0.50 state changes/year) and to Blacks (1.17 state changes/year) (Supplemental Table 3S). These results suggest that Hispanics are the most vulnerable population in structuring transmission clusters, acting as a recipient of viral transmission as well as a source. This result was further supported by our supplemental analysis of grouping Whites, Asians, and Multi-race categories into a single category in a single discrete race/ethnicity condensed model (Supplemental Table 4S).

#### A single discrete race/ethnicity plus age model

When the categories were broken down by age groups, we observed decisively supported (BF > 100) transitions from older (born in or before 1990) Hispanics and older Whites/Others (born in or before 1990) (Fig. 3C, Supplemental Table 5S). There was decisive support for both directions of transmission, with the actual rate as higher from older Whites/Others to older Hispanics than from older Hispanics to older Whites/Others (3.02 and 1.89, respectively). Decisively supported transition rates within a racial/ethnic group from older (born in or before 1990) to younger (born after 1990) groups were observed in Hispanic and Black racial/ethnic groups. The rate of transition from older Blacks to younger Blacks was 1.56. Other decisively supported transition rates were observed from younger Blacks to younger Hispanics (1.55 state transitions/year), younger Hispanics to older Whites/Others (1.50 state transitions/year), and older Whites/Others to younger Whites/Others (0.84 state transitions/year). More detailed findings on actual transition rates and corresponding BFs for a single discrete race/ethnicity model (Supplemental Table 3S), a single discrete race/ethnicity condensed model (Supplemental Table 4S), and a single discrete race/ethnicity plus age model (Supplemental Table 5S) are provided in the online supplemental materials.

#### A single discrete transmission risk model

We observed decisively significant (BF > 100) transitions from MSM to PWID (1.02 state transitions/year) and MSM to cisgender men (1.82 state transitions/year) (Supplemental Figure 1S and Supplemental Table 6S).

### Generalized estimating equation (GEE) analysis

Table 1 presents a summary of estimated population averaged incident rate ratios (*IRRs*) for the assortative mixing (on-diagonals) and disassortative mixing (off-diagonals) terms based on racial-ethnic and age characteristics of the index person (rows) and partners (columns).

A majority of disassortative mixing terms had significant positive associations with the connectivity level at *p* < 0.05. In relation to the earlier phylogenetic findings, all of the decisively supported patterns of transitions that we observed in the single discrete race/ethnicity plus age model also were statistically significant (*p* < 0.001) in their corresponding effects of the index-alter disassortative mixing patterns. The complete statistical analysis results are presented in Supplemental Table 7S in the supplemental materials.

**Table 1:** Summary of estimates of incident rate ratios, using the population averaged negative binomial model to predict network degree (connectivity level) for cluster size ≥ 5 (*N* = 1517 with number of clusters = 143), 2010–2018: phylogenetic links based on assortative mixing (diagonals) and disassortative mixing (off-diagonals).

Population	Number of partners							
	Younger Blacks	Older Blacks	Younger Hispanics	Older Hispanics	Younger Whites	Older Whites	Younger Asians	Older Asians
Younger Blacks	1.14***	1.12***	1.14***	1.15***	1.02	1.14***	1.12	0.82
Older Blacks	1.11***	1.14***	1.18***	1.14***	0.93	1.14***	1.04	1.03
Younger Hispanics	1.11***	1.15***	1.15***	1.12***	1.20***	1.11***	1.14^†^	1.12
Older Hispanics (Index)	1.06^†^	1.15***	1.14***	1.11***	1.20***	1.14***	1.15	1.16**
Younger Whites	1.01	1.30***	1.19***	1.21***	1.19***	1.19***	1.44***	1.13
Older Whites	1.09	1.11***	1.15***	1.10***	1.17***	1.18***	1.13	1.08
Younger Asians	1.04	1.16***	1.15***	1.11^†^	1.23*	1.17***	1.37	1.21
Older Asians	1.04	1.16***	1.15***	1.08***	1.19***	1.20***	1.45**	0.77^†^

Model controls for all study variables listed in Supplemental Table 1S and year of diagnosis. Complete estimated results are provided in Supplemental Table 7S in the online supplemental materials. Asians includes other racial categories of American Indians or Alaskan Natives and Multi-race. Younger is defined as born after 1990, and Older is defined as born in or before 1990. Exchangeable correlation structure and robust variance estimates were used.

^†^*p* < 0.1, *p < 0.05, **p < 0.01, ***p < 0.001.

## Discussion

HIV-1 phylogenetic clusters in Houston/Harris County are characterized by subpopulations who are members of racial/ethnic minority groups. In particular, Hispanic and Latino populations are particularly vulnerable to HIV viral transmission dynamics, increasing the HIV infection disparities due to structural drivers (e.g., HIV stigma in Hispanic/Latino communities, treatment accessibility/availability) that underlie the increase in new HIV infections among these populations^28^. Our study indicates that Hispanic/Latino individuals often serve as the source and recipient of HIV-1 transmission across racial/ethnic groups in Houston. These viral transmission patterns are further characterized by younger Hispanic individuals’ being clustered with older Hispanic individuals at a high connectivity level. A similar pattern was observed among Black individuals. These findings are supported by a recent study based on the U.S. National HIV Surveillance System that reports the most substantial increases in linkage of older MSM to young MSM for Hispanic/Latinos, followed by Blacks, during 2009–2016^29^.

Future research would benefit from an exploration of the effects of changes in the transmission clustering patterns in contexts where services differ in availability to these populations. Access to services, such as HIV testing, HIV medical care, and pre-exposure prophylaxis (PrEP) are of particular importance. Increased HIV testing can facilitate a linkage to HIV medical care for those who are positive and a linkage to PrEP for those who are negative, both of which can blunt the spread of HIV. Viral suppression through adherence to HIV treatment can effectively decrease onward sexual transmission to zero—often referred to as “treatment as prevention”^30^. PrEP is a highly effective pill that is associated with a decreased risk of acquiring HIV for populations at increased HIV infection risk, although the effectiveness level depends on the level of adherence^31^. A recent study demonstrated that increases in PrEP coverage, regardless of changes in viral suppression, are significantly associated with decreases in HIV diagnosis rates^32^, a finding that substantiates the need to increase service availability among those most vulnerable.

HIV-1 transmission cluster patterns that involve racial/ethnic minority groups are complex and multifaceted in terms of cluster membership, size, and connectivity within clusters, as they are inextricably associated with other demographic, transmission risk, and social/cultural factors. For instance, younger Black and Hispanic MSM tend to have other transmission partners similar in age^33^. Of note, the Hispanic and Latino culture in the U.S. is diverse, and regional differences exist in the predominant mode of transmission and associated HIV transmission risks^34^. Cluster membership and size also are further complicated by the way that other factors, such as immigration status and country of origin, interact in the transmission cluster^35,36^. For example, among Hispanic immigrants, cluster membership is associated with younger ages, more acculturation to the U.S., and being of Mexican origin^35^. Further, larger clusters that involve Hispanic members tend to contain fewer immigrants and more MSM, and Hispanic immigrants tend to be in clusters with other Hispanics^35^, indicating their having putative U.S.-born transmission partners^36^.

Given the racial/ethnic and cultural diversity in the Houston area, our study findings provide a unique perspective to investigating the HIV-1 clustering patterns in local HIV-1 transmission networks. Houston, in addition to being the largest city in the Southern U.S.^37^, is one of the fastest growing cities and has one of the youngest populations in the U.S., due mainly to the influx of immigrants^38^. In Harris County (which encompasses much of Houston), Hispanics are the plurality and, when combined with Black and other minority racial/ethnic groups, comprise 68.7% of the total population^39^.

Our study findings are constrained by some limitations. Our sample came from the first sequence test available for each person, which may exclude those who remained virally unsuppressed, which limits our ability to identify more of the clustered individuals when using multiple sequences per person in identifying clusters^40^. In addition, our sample accounts only for individuals who have been diagnosed and have had a sequence of good quality reported to the health department. Our study may have been limited in reconstructing closely related viral transmission clusters due to potential missing sequences from an intermediary or a common source of a pairwise, similarly-strained virus. A more complete reconstruction of the HIV-1 transmission networks could be realized by combining an epidemiological linkage via contact tracing of risk-sharing partners^41^, social network members^42^, or online or physical venue affiliation^2,43^. Future studies would likely benefit from combining molecular, epidemiological, and social network data to identify growing clusters and by considering the geographic proximity that may be associated with clustering patterns^6,9,44^. Further, HIV is known to be disproportionately transmitted by recently infected individuals^45^ and transmitted at an acute stage of HIV infection^9,46^. HIV-1 clustering has been reported to be associated with clinical factors, such as higher HIV viral load^8,44,46^, lack of antiretroviral use^44^, and higher CD4^+^ T-cell counts^10,44^. Future phylodynamic research also could consider these clinical factors in defining the epidemiological profile associated with the dynamics of HIV-1 transmission patterns. Finally, our statistical approach has limitations in dealing with potential autocorrelation of the error term and addressing network dependency of phylogenetic links. Future research could use exponential random graph models to estimate assortative mixing patterns with controls of the dependent nature of the HIV-1 transmission network by partitioning it into distinct neighborhoods^47^.

Despite these limitations, our study provides a unique opportunity to chart the dynamics of HIV-1 transmission between different groups by race/ethnicity, age, and transmission risk in the racially/ethnically diverse context of Houston, TX. The intensified transmission rates early in HIV infections have been attributed to the growth of larger clusters^48,49^. Our findings are consistent with the recommendation of the routine use of HIV sequence data to interrupt HIV transmission through prevention interventions, with the aim of preventing the onward transmission of HIV-1 among these vulnerable younger racial/ethnic minorities, in particular, younger Hispanic subpopulations.

## Supplementary Information

Supplementary Information.
