# Author Correction: Methodological synthesis of Bayesian phylodynamics, HIV-TRACE, and GEE: HIV-1 transmission epidemiology in a racially/ethnically diverse Southern U.S. context

**DOI:** 10.1038/s41598-021-89879-w

**Published:** 2021-05-10

**Authors:** Kayo Fujimoto, Justin Bahl, Joel O. Wertheim, Natascha Del Vecchio, Joseph T. Hicks, Lambodhar Damodaran, Camden J. Hallmark, Richa Lavingia, Ricardo Mora, Michelle Carr, Biru Yang, John A. Schneider, Lu-Yu Hwang, Marlene McNeese

**Affiliations:** 1 Department of Health Promotion and Behavioral Sciences, The University of Texas Health Science Center At Houston, 7000 Fannin Street, UCT 2514, Houston, TX 77030 USA; 2 Department of Infectious Diseases, University of Georgia, Athens, GA USA; 3 Department of Medicine, University of California San Diego, La Jolla, CA USA; 4 Department of Biostatistics and Data Science, The University of Texas Health Science Center at Houston, Houston, TX USA; 5 Institute of Bioinformatics, University of Georgia, Athens, GA USA; 6 Division of Disease Prevention and Control, Houston Health Department, Houston, TX USA; 7 Department of Medicine, University of Chicago, Chicago, IL USA; 8 Department of Epidemiology, Human Genetics, and Environmental Science, The University of Texas Health Science Center at Houston, Houston, TX USA

Correction to: *Scientific Reports* 10.1038/s41598-021-82673-8, published online 08 February 2021

The Acknowledgements section in this Article is incomplete.

“We acknowledge R. Lubelchek, J. Xu, M. Imahashi, W. Zhou, N.-W. Sheung, M. Matsuda, A. Hochi, and individuals from the following areas of the Houston Health Department: Informatics Program, HIV Surveillance Program, and the Bureau of HIV/STD and Viral Hepatitis Prevention.”

should read:

“We acknowledge R. Lubelchek, J. Xu, M. Imahashi, W. Zhou, N.-W. Sheung, M. Matsuda, A. Hochi, and individuals from the following areas of the Houston Health Department: Informatics Program, HIV Surveillance Program, and the Bureau of HIV/STD and Viral Hepatitis Prevention. The contents of this publication are solely the responsibility of the authors and do not necessarily represent the official views of the National Institutes of Health, the Centers for Disease Control and Prevention, or the Department of Health and Human Services.”

Furthermore, the Funding section in this Article contains typographical errors, where

“This work was supported by the Centers for Disease Control and Prevention (CDC) (Grants 5 NU62PS924515-03-00 and 1 NU62PS924572-01-00 to M.M.) and the National Institutes of Health (NIH) (Grants 1R21GM113694 to K.F., 1R01MH100021 to K.F. and J.A.S., R01AI136056 to J.A.S., K01AI110181 to J.O.W., R01AI135992 to J.O.W., and 1R56AI150272-01A1 to K.F and J.A.S.).”

should read:

“This work was supported by the Centers for Disease Control and Prevention (CDC) (Cooperative Agreements NU62PS924515 and NU62PS924572 to M.M.) and the National Institutes of Health (NIH) (Grants 1R21GM113694 to K.F., 1R01MH100021 to K.F. and J.A.S., R01AI136056 to J.A.S., K01AI110181 to J.O.W., R01AI135992 to J.O.W., and 1R56AI150272-01A1 to K.F and J.A.S.).”

