# Supplementary material for: Methodological synthesis of Bayesian phylodynamics, HIV-TRACE, and GEE: HIV-1 transmission epidemiology in a racially/ethnically diverse Southern U.S. context
Source: Sci Rep. 2021 Feb 8;11:3325. doi: 10.1038/s41598-021-82673-8 (PMC7870963; doi:10.1038/s41598-021-82673-8)
Supplement: Supplementary file 1 — Supplementary Information. [file 41598_2021_82673_MOESM1_ESM.pdf]

Supplemental Digital Content (SDC)

Title: Methodological synthesis of Bayesian phylodynamics, HIV-TRACE, and GEE: HIV-1 transmission epidemiology in a racially/ethnically diverse Southern U.S. context

Kayo Fujimoto, PhD[a*]
Justin Bahl, PhD[b*]
Joel O. Wertheim, PhD[c]
Natascha Del Vecchio, MS[d]
Joseph Hicks, DVM, PhD[b]
Lambodhar Damodaran, BS[e]
Camden J. Hallmark, MPH[f]
Richa Lavingia, MPH[a]
Ricardo Mora, MPH[f]
Michelle Carr, MPH[f]
Biru Yang, PhD, MPH[f]
John A. Schneider, MD, MPH[g]
Lu-Yu Hwang, MD[h]
Marlene McNeese, BS[f]

[a]Department of Health Promotion and Behavioral Sciences, University of Texas Health Science Center at Houston, Houston, TX

[b]Department of Infectious Diseases, University of Georgia, Athens, GA

[c]Department of Medicine, University of California San Diego, La Jolla, CA

[d]Department of Biostatistics and Data Science, University of Texas Health Science Center at Houston, Houston, TX

[e]Institute of Bioinformatics, University of Georgia, Athens, GA

[f]City of Houston Health Department, Houston, TX

[g]Department of Medicine, University of Chicago, Chicago, IL

[h]Department of Epidemiology, Human Genetics, and Environmental Science, University of Texas Health Science Center at Houston, Houston, TX

Abbreviations: BEAST, Bayesian Evolutionary Analysis Sampling Trees; BF, Bayes Factor; BSSVS, Bayesian Stochastic Search Variable Selection; GTR, general time-reversible; HIV-TRACE, HIV-TRAnsmission Cluster Engine; IDU, injection drug use; *IRR*, incident rate ratio (estimated population averaged incident rate ratios); MSM, men who have sex with men; PWID, people who inject drugs.

Descriptive Statistics for Characteristics of Individuals

Table 1S presents descriptive statistics for characteristics of Individuals. A majority of the 6,332 individuals in our sample were older (born in or before 1990) (81%), males (80%), of a racial/ethnic minority (47% Blacks and 36% Hispanics), and MSM (54%). In addition, 42% had an initial viral load after HIV diagnosis greater than 10,000 copies/ml, and 88% had a $CD4^+$ T-cell count of > 200. As for *Cluster identification.* We identified 2,675 (42%) samples with a cluster size of two or more out of 6,332 samples, with a mean cluster size of 12 (SD = 14.2, min = 2, max = 58). Among these 2,675 samples, 1,529 (57%) belonged to 143 unique clusters, with five or more members with a mean cluster size of 18.5 (SE = 15.7, min = 5, max = 58). The categories of younger individuals (38%), males (27%), Whites (27%), MSM (31%), viral load of 10,000–100,000 (26%), and $CD4^+$ T-cell count of >350 (28%) showed a higher percentage of membership within a cluster that comprised ≥ 5 members.

Table 1S. Descriptive Statistics for Characteristics of Individuals in Cluster Sizes ≥ 5 and Connectivity (Network Degree) with Significance Test: Frequencies (Percentages) and Means (Standard Deviations, Minimum, Maximum Values) (*N* = 6,332), 2010–2018.

Study variable	Total	Cluster sizes (*n* ≥ 5) Yes (*N* = 1,529)	No (*N* = 4,803)	*p-value*	Network degree	*p-value*
Age				0.001		<0.10
Younger	1,183 (18.7%)	450 (38.0%)	733 (62.0%)		7.3 (6.5, 0, 37)	
Older	5,149 (81.3%)	1,079 (21.0%)	4,070 (79.0%)		6.8 (6.5, 1, 42)	
Sex assigned at birth				0.001		<0.001
Male	5,036 (79.5%)	1,351 (26.8%)	3,685 (73.2%)		7.3 (6.7, 1, 42)	
Female	1,296 (20.5%)	178 (13.7%)	1,118 (86.3%)		4.5 (3.9, 1, 24)	
Race/ethnicity				<0.05		<0.01
Hispanics	2,254 (35.6%)	579 (25.7%)	1,675 (74.3%)		7.5 (6.7, 1, 42)	
Blacks	2,949 (46.6%)	655 (22.2%)	2,294 (77.8%)		6.5 (6.5, 1, 38)	
Whites	866 (13.7%)	233 (26.9%)	633 (73.1%)		6.7 (5.6, 1, 35)	
Asians/Others	103 (1.6%)	25 (24.3%)	78 (75.7%)		9.2 (9.6, 1, 33)	
Multi-race	160 (2.5%)	37 (23.1%)	123 (76.9%)		6.6 (6.2, 1, 27)	
CDC transmission category				<0.001		<0.001
MSM	3,410 (53.9%)	1,063 (31.2%)	2,347 (68.8%)		7.5 (6.9, 1, 42)	
Cisgender women	1,250 (19.7%)	170 (13.6%)	1,080 (86.4%)		4.5 (4.0, 1, 24)	
Cisgender men	1,352 (21.4%)	217 (16.0%)	1,135 (84.0%)		5.9 (5.2, 1, 31)	
Transgender women	84 (1.3%)	23 (27.4%)	61 (72.6%)		7.7 (8.5, 1, 32)	
PWID	236 (3.7%)	56 (23.7%)	180 (76.3%)		7.4 (6.9, 1, 27)	
Viral load				<0.001		>0.10
<10,000	1,402 (22.1%)	285 (20.3%)	1,117 (79.7%)		6.9 (7.0, 1, 38)	
10,000–100,000	2,707 (42.8%)	709 (26.2%)	1,998 (73.8%)		6.8 (6.4, 1, 42)	
>100,000	2,223 (35.1%)	535 (24.1%)	1,688 (75.9%)		7.2 (6.4, 1, 40)	
$CD4^+$ T-cell count				<0.001		>0.1
<0	720 (11.4%)	87 (12.1%)	633 (87.9%)		6.1 (4.8, 1, 33)	
50–200	1,054 (16.6%)	173 (16.4%)	881 (83.6%)		6.6 (6.4, 1, 42)	
201–350	1,359 (21.5%)	368 (27.1%)	991 (72.9%)		7.2 (6.7, 1, 37)	
>350	3,148 (49.7%)	893 (28.4%)	2,255 (71.6%)		7.0 (6.7, 1, 38)	

Note: *p*-values were computed based on the chi-square test for cluster membership in cluster size ≥ 5 for categorical variables. For the level of connectivity (network degree) between categorical variables, the Wilcoxon rank-sum (Mann-Whitney) test and Kruskal-Wallis equality-of-populations rank test were used for cluster size ≥ 5. MSM refers to cisgender men who report being MSM, Cisgender women refers to cisgender women who did not report IDU, Cisgender men refers to cisgender men who did not report IDU or MSM, Transgender women refers to transgender women who did not report IDU, and PWID refers to anyone who reports IDU, including MSM. The sample consists of 838 (13%) from 2010, 791 (12%) from 2011, 854 (13%) from 2012, 812 (13%) from 2013, 796 (13%) from 2014, 760 (12%) from 2015, 747 (12%) from 2016, 484 (8%) from 2017, and 250 (4%) from 2018. Information on missing values are as follows: 51 (0.81%) for CD4 counts. Younger is defined as born after 1990; older is defined as born in or before 1990.

Table 2S. Partners for Cluster Size ≥ 5

Population	Mean (*SD*, minimum maximum)
Younger Hispanics	0.8 (1.5, 0, 8)
Younger Blacks	1.1 (2.2, 0, 16)
Younger Whites	0.2 (0.5, 0, 6)
Younger Asians/Others	0.1 (0.3, 0, 2)
Older Hispanics	2.1 (3.2, 0, 27)
Older Blacks	1.7 (2.7, 0, 19)
Older Whites	0.9 (1.5, 0, 10)
Older Asians/Others	0.2 (0.4, 0, 3)

Phylogenetic Analytic Design

Our phylogenetic analysis design followed the three-step process of: (1) identification of transmission clusters, (2) estimation of the time of most recent common ancestor for each cluster, and (3) joint estimation of a single viral transition matrix for all clusters. For Steps 2 and 3, our phylogenetic analysis was based on the use of clusters with five or more members and the exclusion of homogeneous clusters for computational tractability and the ability to infer rates that were meaningful and realistic across all identified clusters.

For Step 1, we constructed a genetic transmission network, using HIV-TRACE.[1, 2] All sequences were aligned to an HXB2 reference sequence (positions 2253–3749). To identify genetically linked pairs, we used a pairwise genetic distance threshold of 0.015 substitutions/site and an ambiguity fraction of 1.5%.

In Step 2, we estimated a preliminary maximum likelihood phylogenetic tree through the use of a GTR model with gamma distributed rate variation among sites (GTR Gamma), using RAxML v8.2.4.[3] The “clocklikeness” of the tree was determined using TempEst v.1.5.3 with a root-to-tip linear regression.[4] Then, we conducted statistical phylodynamic analysis, using

BEAST v 1.10.1.[5] All sequences were aligned to generate a rooted, time-measured phylogeny inferred using a relaxed molecular clock model to estimate the time to the most recent common ancestor of each cluster. We used the GTR 4 substitution model that accounts for the proportion of invariant sites that do not undergo evolutionary change. We specified a lognormal uncorrelated relaxed molecular clock model and a time-aware Gaussian Markov Random Field Skyride coalescent tree prior. Then, we performed six independent Markov chain Monte Carlo runs of 100 million states in length, sampling every 10,000 states.[6] We examined the logs, using Tracer 1.7.1, combined with the use of LogCombiner 1.10.4 after the removal of an appropriate burn-in, and generated a maximum clade credibility (MCC) tree, using TreeAnnotator 1.10.4. We recorded the time to the most the recent common ancestor of each cluster and used an informative prior to generate phylogenies of each independent cluster with the same conditions described above. Parameter estimations were summarized from three independent Markov chain Monte Carlo runs of 10 million states in length, sampling every 1,000 states. We used Tracer v1.7.1 to assess the convergence and specify the appropriate burn-in for each run.[7]

In Step 3, to assess the importance of epidemiological parameters in HIV transmission, we performed an ancestral state reconstruction[8] of racial/ethnic and transmission risk characteristics, using Bayesian Stochastic Search Variable Selection (BSSVS). For phylodynamic analysis, we used clusters constructed using a more conservative genetic distance threshold of 0.005 substitutions/site. This lower threshold will identify more recent potential transmission partners[9] with fewer intermediate partners. Intermediate partners can confound estimates of frequencies of transmission among risk groups.[10] We estimated a single discrete state transition rate matrix jointly across all clusters independently instead of one matrix for a

tree that contained all clusters under the assumption that underlying characteristics are shared by all clusters in Houston/Harris County.[11]

We jointly estimated a single phylodynamic discrete trait model to all clusters for each trait of race/ethnicity and transmission risk. We used this joint estimation approach because epidemiological characteristics of a population can determine the structure of outbreaks. Individual clusters, however, are linked on a phylogenetic tree by long branches. These branches represent unobserved members of an outbreak, viral migration, or a biological change that triggered the outbreak. Phylodynamic approaches to hypothesis testing, including ancestral state reconstruction of tip-associated discrete traits, assume that the rate of change is constant throughout the branches of the phylogenetic tree. Although this assumption is reasonable for individual clusters, it is unlikely to be valid for the entire tree. Therefore, a jointly fitted single discrete model should capture the signal of the population's social structure that may determine patterns of HIV transmission.

The tip-associated traits that we analyzed included race/ethnicity, race/ethnicity plus year of birth (born in or before 1990 and after 1990), and transmission risk. We used an asymmetric continuous-time Markov chain model[12] for discrete state reconstructions and performed three independent Markov chain Monte Carlo runs with a chain length of 10 million states, logging every 1,000 for each model. We applied ancestral state reconstruction of a racial/ethnicity discrete state model to an empirical set of 1,500 phylogenetic trees for each cluster.

Given the large number of states, BSSVS was employed to search parameter space and identify those parameters with significantly non-zero transition rates.[8, 12] BSSVS explores and efficiently reduces the state space by employing a binary indicator (I).[8, 12] From the BSSVS results, we applied a Bayes factor (BF) test to assess the support for individual transitions

between discrete states. We removed a burn-in of 10% (100,000) and computed BF values, using SpreaD3.[13] We used the following scheme to assess the level of support: No support: BF < 3; substantial support: BF = 3–10; strong support: BF = 11–30; very strong support: BF = 31–100; decisive support: BF > 100.

Results of Transition Rate Matrix for A Single Discrete Race/Ethnicity Model

Table 3S presents transition rates (A) and corresponding BF test results (B) for a single discrete race/ethnicity model. In this analysis, other racial categories were excluded, as there were only two sequences that represent American Indians or Alaskan Natives, and they did not cluster into a group that met the criteria for inclusion.

Table 3S. Transition Rate Matrix for Single Discrete Race/Ethnicity Model.

(A) Rates for a Single Discrete Race/Ethnicity Model

	Recipient				
Source	Hispanics	Asians	Blacks	Multi-race	Whites
Hispanics	--	0.50	1.17	0.26	1.84
Asians	0.58	--	0.42	0.25	0.43
Blacks	2.55	0.13	--	0.47	0.49
Multi-race	0.39	0.24	0.35	--	0.47
Whites	2.99	0.25	0.53	0.29	--

Note: Asians include other racial/ethnic groups. Rates range from 0.13 to 2.99.

(B) Bayes Factor for a Single Discrete Race/Ethnicity Model

	Recipient				
Source	Hispanics	Asians	Blacks	Multi-race	Whites
Hispanics	--	3,055	6,647	>1	153,011
Asians	2	--	>1	>1	<1
Blacks	153,011	>1	--	78	5
Multi-race	2	>1	2	--	4

Whites	153,011	>1	2	43	--

Note: Asians include other racial/ethnic groups. Rates range from 0.13 to 2.99. Bayes factor support: 3–10 = substantial support, 11–30 = strong support, 31–100 = very strong support, >100 = decisive support.

We identified 34 racially/ethnically non-homogeneous transmission clusters. We used an asymmetric model to allow for the directionality of transmission to be estimated. We observed decisively significant (BF > 100) transitions from Whites to Hispanics (2.99 state changes/year), from Blacks to Hispanics (2.55 state changes/year), and from Hispanics to Whites (1.84 state changes/year). Further, Hispanics had decisively supported transitions to Asians (0.50 state changes/year) and to Blacks (1.17 state changes/year). These results suggest that Hispanics are the most vulnerable population in structuring transmission clusters, acting as a recipient of viral transmission as well as a source.

Results of Transition Rate Matrix for A Single Discrete Race/Ethnicity Condensed Model

Table 4S presents transition rates (A) and corresponding BF test results (B) for a single discrete condensed race/ethnicity model. In this condensed model, we grouped the White, Asian, and Multi-race categories into a single category of Whites/Others and excluded other racial categories of American Indians or Alaskan Natives. We observed decisively significant transitions from Whites/Others to Hispanics (2.05 state transitions/year), from Blacks to Hispanics (1.43 state transitions/year), and from Hispanics to Whites/Others (1.57 state transitions/year).

Table 4S. Transition Rate Matrix for a Single Discrete Race/Ethnicity Condensed Model.

(A) Rates for a Single Discrete Race/Ethnicity Condensed Model

	Recipient		
Source	Hispanics	Blacks	White/Others
Hispanics	--	0.84	1.57
Blacks	1.43	--	0.48
White/Others	2.05	0.34	--

Note: Rates range from 0.34 to 2.05.

(B) Bayes Factor for a Single Discrete Race/Ethnicity Condensed Model

	Recipient		
Source	Hispanics	Blacks	White/Others
Hispanics	--	2,249	27,002
Blacks	27,002	--	8
White/Others	27,002	1	--

Note: Bayes factor support: 3–10 = substantial support, 11–30 = strong support, 31–100 = very strong support, >100 = decisive support.

Results of Transition Rate Matrix for a Single Discrete Race/Ethnicity Plus Age Model

Table 5S presents the transition rates (A) and corresponding BF test results (B) for a single discrete race/ethnicity plus age model.

Table 5S. Transition Rate Matrix for a Single Discrete Race/Ethnicity Model Plus Age Model.

(A) Rates for a Single Discrete Race/Ethnicity Plus Age Model

	Recipient					
Source	Hispanics-Pre	Blacks-Pre	Whites/Others-Pre	Hispanics-Post	Blacks-Post	Whites/Others-Post
Hispanics-Pre	--	0.87	1.89	1.64	0.79	0.53
Blacks-Pre	1.03	--	0.70	0.68	1.56	0.34
Whites/Others-Pre	3.02	0.87	--	0.60	0.44	0.84

Hispanics-Post	0.86	0.89	1.50	--	0.46	0.80
Blacks-Post	1.07	1.36	0.60	1.55	--	0.41
Whites/Others-Post	1.32	0.48	0.74	0.74	0.48	--

Note: Pre = born in or before 1990, Post = born after 1990. Rates range from 0.44 to 3.02.

(B) Bayes Factor for a Single Discrete Race/Ethnicity Plus Age Model

	Recipient					
Source	Hispanics-Pre	Blacks-Pre	Whites/Others-Pre	Hispanics-Post	Blacks-Post	Whites/Others-Post
Hispanics-Pre	--	25	243,018	8,671	5	6
Blacks-Pre	25	--	30	4	1,305	2
Whites/Others-Pre	243,018	19	--	2	2	508
Hispanics-Post	5	16	664	--	32	55
Blacks-Post	22	86	5	5,392	--	1
Whites/Others-Post	23	2	4	4	2	--

Note: Pre = born in or before 1990, Post = born after 1990. Bayes factor support: 3–10 = substantial support, 11–30 = strong support, 31–100 = very strong support, >100 = decisive support.

When broken down by age groups, we observed decisively supported transitions from older (born in or before 1990) Hispanics and older Whites/Others (born in or before 1990). There was strong support for both directions of transmission, with the rate as higher from older Whites/Others to older Hispanics than from older Hispanics to older Whites/Others (3.02 and 1.89, respectively). Decisively supported transition rates within a racial/ethnic group from older (born in or before 1990) to younger (born after 1990) groups were observed in Hispanic and Black racial/ethnic groups. The rates of transition from older Hispanics to younger Hispanics was 1.64. Rates of transition from older Blacks to younger Blacks was 1.56. Other decisively supported transition rates observed were from younger Blacks to younger Hispanics (1.55 state transitions/year), younger Hispanics to older Whites/Others (1.50 state transitions/year), and older Whites/Others to younger Whites/Others (0.84 state transitions/year). Taken together, our

results suggest that critical interventions that might limit transmission are not reaching the Hispanic community in Houston.

Results of Transition Rate Matrix for a Single Discrete Transmission Risk Model

Table S6 presents transition rates (A) and corresponding BF test results (B) for a single discrete transmission risk model.

Table 6S. Single Discrete Transmission Risk Model.

(A) Rates for a Single Discrete Transmission Risk Model

	Recipient			
Source	PWID	MSM	Cis-women	Cis-men
Hispanics	--	1.80	0.58	0.46
Asians	1.02	--	0.43	1.82
Blacks	0.55	1.00	--	1.00
Multi-race	0.44	1.86	0.89	--

Note: MSM = cisgender men who report being MSM, cis-women refers to cisgender women who did not report IDU, cis-men refers to cisgender men who did not report IDU or MSM, PWID refers to anyone who reports IDU, including MSM. Rates range from 0.43 to 1.86.

(B) Bayes Factor for a Single Discrete Transmission Risk Model

	Recipient			
Source	PWID	MSM	Cis-women	Cis-men
Hispanics	--	11	2	1
Asians	81,006	--	44	81,006
Blacks	9	3	--	11
Multi-race	1	10	4	--

Note: MSM = cisgender men who report being MSM, cis-women refers to cisgender women who did not report IDU, cis-men refers to cisgender men who did not report IDU or MSM, PWID refers to anyone who reports IDU, including MSM. Bayes factor support: 3–10 = substantial support, 11–30 = strong support, 31–100 = very strong support, >100 = decisive support.

We identified 27 non-homogeneous clusters with respect to transmission risk categories with ≥ 5 members. We observed decisively significant transitions from MSM to PWID (1.02

state transitions/year, BF >100) and MSM to cisgender men (1.82 state transitions/year, BF >100). Our analysis indicates marginal support (BF = 3–10) for the transitions from PWID to MSM (1.80 state transitions/year, BF = 11) and from cisgender women to cisgender men (1.00 state transition/year, BF = 11). These results, however, should be interpreted with the caveat that potential sampling bias and lack of accurate transmission risk information may obscure the true signal. In some cases, transmission risk categories were poorly represented and were removed or re-categorized (e.g., an un-clustered transgender man was excluded from the analysis; transgender women also were excluded from three clusters). In addition, there is the potential for the cisgender men category to contain MSM, which was not indicated and who were coded as cisgender men due to being a male and non-reporting or having no risk indicated.

Results of BEAST Phylogeny from both Race/Ethnicity and Transmission Risk HIV Cluster Analysis

Figure S1 indicates that there were no frequently observed patterns or significant groupings regarding the race/ethnicity or transmission risk categories for the taxa.

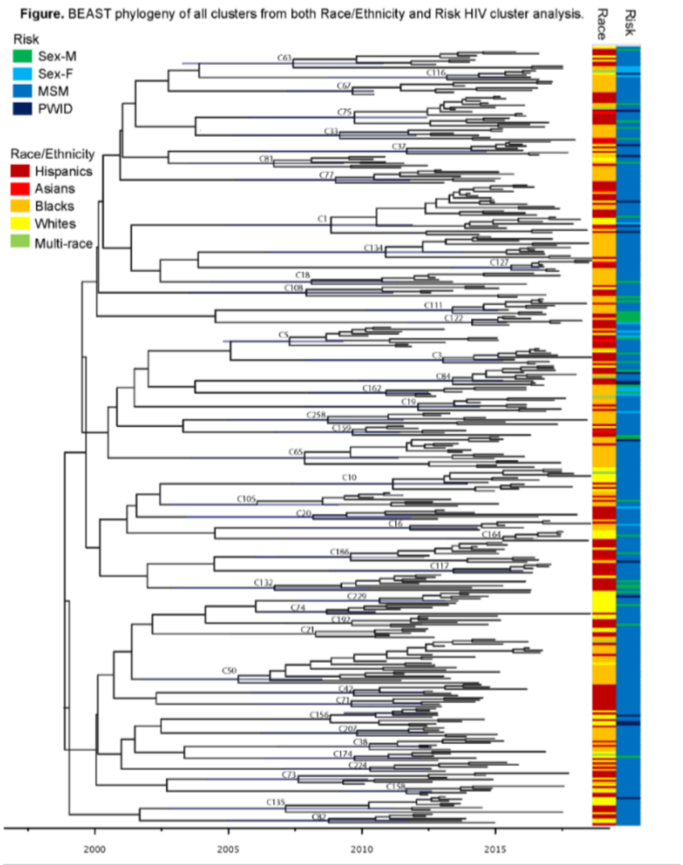

Figure 1S. BEAST Phylogeny of All Clusters from both Race/Ethnicity and Transmission Risk.

Results of Generalized Estimating Equations

Table 7S presents estimated results of our population-averaged negative binomial model for network degree (connectivity level) (N = 1,529 with number of clusters = 143) for cluster size of 5 or more.

Table 7S. Incidence Rate Ratios, Using the Population Averaged Negative Binomial Model to Predict Network Degree (Connectivity Level) for Cluster Size ≥ 5 (*N* = 1,529 with the number of clusters = 143), 2010–2018: Assortative Mixing (diagonals), and Disassortative Mixing (off-diagonals) Phylogenetic Links.

**Study variable**	**IRR**	**SE**	**95% CIs**	
Age				
Younger (Born after 1990)	0.75*	0.08	0.60	0.94
Older (Born in or before 1990)		Reference		
Race/Ethnicity	0.93	0.07	0.81	1.08
Blacks				
Hispanics	0.99	0.05	0.89	1.09
Asians/Others	0.95	0.06	0.83	1.09
Whites		Reference		
CDC Transmission Category				
PWID	0.95	0.04	0.87	1.03
Transgender women	1.00	0.07	0.87	1.15
Cisgender women	0.99	0.03	0.94	1.05
Cisgender men	1.03†	0.02	1.00	1.07
MSM		Reference		
Logged viral loads	1.00	0.00	0.99	1.01
$CD4^+$ T-cell count				
> 350	0.96	0.03	0.90	1.02
201–350	0.96	0.03	0.90	1.02
50–200	0.96	0.03	0.90	1.02
< 50		Reference		
Younger Blacks	1.33**	0.16	1.06	1.68
Younger Hispanics	1.27†	0.16	1.00	1.61
Younger Asians/Others	1.37	0.38	0.79	2.36
Assortative mixing (index – partner matching terms)				
Younger Blacks – Younger Black partners	1.14***	0.02	1.10	1.18
Older Blacks – Older Black partners	1.14***	0.02	1.10	1.18
Younger Hispanics – Younger Hispanic partners	1.15***	0.01	1.13	1.18
Older Hispanics – Older Hispanic partners	1.11***	0.03	1.05	1.16
Younger Whites – Younger White partners	1.21***	0.03	1.15	1.26
Older Whites – Older White partners	1.18***	0.02	1.14	1.21
Younger Asians/Others – Younger Asians/Other partners	1.37	0.40	0.77	2.44
Older Asians/Others – Older Asians/Other partners	0.77†	0.12	0.57	1.05
Disassortative mixing (index – partner mismatching terms)				
Younger Blacks – Older Black partners	1.12***	0.02	1.08	1.16
Younger Blacks – Younger Hispanic partners	1.14***	0.02	1.10	1.19
Younger Blacks – Older Hispanic partners	1.15***	0.03	1.08	1.21
Younger Blacks – Younger White partners	1.02	0.13	0.79	1.32

Younger Blacks – Older White partners	1.14***	0.04	1.06	1.22
Younger Blacks – Younger Asians/Other partners	1.12	0.10	0.94	1.33
Younger Blacks – Older Asians/Other partners	0.82	0.12	0.63	1.09
Older Blacks – Younger Black partners	1.11***	0.03	1.06	1.17
Older Blacks – Younger Hispanic partners	1.18***	0.04	1.10	1.26
Older Blacks – Older Hispanic partners	1.14***	0.03	1.09	1.19
Older Blacks – Younger White partners	0.93	0.07	0.80	1.08
Older Blacks – Older White partners	1.14***	0.02	1.10	1.18
Older Blacks – Younger Asians/Other partners	1.04	0.09	0.87	1.24
Older Blacks – Older Asians/Other partners	1.03	0.07	0.91	1.16
Younger Hispanics – Older Hispanic partners	1.12***	0.02	1.08	1.17
Younger Hispanics – Younger Black partners	1.11***	0.04	1.04	1.18
Younger Hispanics – Older Black partners	1.15***	0.03	1.08	1.21
Younger Hispanics – Younger White partners	1.20***	0.03	1.15	1.25
Younger Hispanics – Older White partners	1.11***	0.03	1.06	1.17
Younger Hispanics – Younger Asians/Other partners	1.14†	0.08	0.99	1.32
Younger Hispanics – Older Asians/Other partners	1.12	0.08	0.97	1.29
Older Hispanics – Younger Hispanic partners	1.14***	0.02	1.10	1.19
Older Hispanics – Younger Black partners	1.06†	0.03	1.00	1.13
Older Hispanics – Older Black partners	1.15***	0.02	1.12	1.18
Older Hispanics – Younger White partners	1.20***	0.03	1.15	1.26
Older Hispanics – Older White partners	1.14***	0.02	1.11	1.17
Older Hispanics – Younger Asians/Other partners	1.15	0.12	0.95	1.41
Older Hispanics – Older Asians/Other partners	1.16***	0.06	1.05	1.29
Younger Whites – Older White partners	1.19***	0.02	1.15	1.23
Younger Whites – Younger Black partners	1.01	0.05	0.92	1.11
Younger Whites – Older Black partners	1.30***	0.07	1.17	1.45
Younger Whites – Younger Hispanic partners	1.19***	0.02	1.15	1.24
Younger Whites – Older Hispanic partners	1.21***	0.04	1.14	1.28
Younger Whites – Younger Asians/Other partners	1.44***	0.13	1.21	1.73
Younger Whites – Older Asians/Other partners	1.13	0.09	0.97	1.32
Older Whites – Younger White partners	1.17***	0.04	1.10	1.25
Older Whites – Younger Black partners	1.09	0.05	0.99	1.20
Older Whites – Older Black partners	1.11***	0.03	1.05	1.16
Older Whites – Younger Hispanic partners	1.15***	0.02	1.12	1.19
Older Whites – Older Hispanic partners	1.10***	0.02	1.06	1.15
Older Whites – Younger Asians/Other partners	1.13	0.12	0.92	1.38
Older Whites – Older Asians/Other partners	1.08	0.09	0.92	1.26
Younger Asians/Others – Older Asians/Other partners	1.21	0.30	0.75	1.95
Younger Asians/Others – Younger Black partners	1.04	0.06	0.92	1.17
Younger Asians/Others – Older Black partners	1.16***	0.04	1.08	1.25
Younger Asians/Others – Younger Hispanic partners	1.15***	0.03	1.10	1.21

Younger Asians/Others – Older Hispanic partners	1.11†	0.06	0.99	1.24
Younger Asians/Others – Younger White partners	1.23*	0.12	1.02	1.49
Younger Asians/Others – Older White partners	1.17***	0.02	1.13	1.21
Older Asians/Others – Younger Asians/Other partners	1.45**	0.16	1.18	1.80
Older Asians/Others – Younger Black partners	1.04	0.04	0.97	1.11
Older Asians/Others – Older Black partners	1.16***	0.03	1.11	1.21
Older Asians/Others – Younger Hispanic partners	1.15***	0.03	1.09	1.22
Older Asians/Others – Older Hispanic partners	1.08***	0.02	1.04	1.13
Older Asians/Others – Younger White partners	1.19***	0.04	1.10	1.27
Older Asians/Others – Older White partners	1.20***	0.03	1.13	1.27

Note: Models control for HIV diagnosis year (2010–2018). Exchangeable correlation structure and robust variance estimates are used. The Asians/Others category includes other racial categories of American Indians or Alaskan Natives and Multi-race. Younger is defined as born after 1990, and Older is defined as born in or before 1990. Exchangeable correlation structure and robust variance estimates were used.
†p< 0.1, *p < 0.05, **p < 0.01, ***p < 0.001 for two-tailed test.

A majority of disassortative mixing terms had significant positive associations with the connectivity level at $p < 0.05$. In relation to the earlier phylogenetic findings, all of the decisively supported patterns of transitions that we observed in the single discrete race/ethnicity plus age model also were statistically significant ($p < 0.001$) in their corresponding effects of the index-alter disassortative mixing patterns, as follows: (1) older Hispanics who have more older White partners (*IRR* = 1.14, *CIs*: 1.11, 1.17), (2) older Whites who have more older Hispanic partners (*IRR* = 1.10, *CIs*: 1.06, 1.15), (3) older Hispanics who have more younger Hispanic partners (*IRR* = 1.14, *CIs*: 1.10, 1.19), (4) older Blacks who have more younger Black partners (*IRR* = 1.11, *CIs*: 1.06, 1.17), (5) younger Blacks who have more younger Hispanic partners (*IRR* = 1.14, *CIs*: 1.10, 1.19), (6) younger Hispanics who have more older White partners (*IRR* = 1.11, *CIs*: 1.06, 1.17), and (7) older Whites who have more younger White partners (*IRR* = 1.17, *CIs*: 1.10, 1.25).

References

1. Pond, S.L.K., Weaver, S., Leigh Brown, A.J. & Wertheim, J.O.. HIV-TRACE (TRAnsmission Cluster Engine): A tool for large scale molecular epidemiology of HIV-1 and other rapidly evolving pathogens. *Mol Biol Evol.* **35**(7), 1812–1819 (2018).

2. Weaver, S. *et al*. Datamonkey 2.0: A modern web application for characterizing selective and other evolutionary processes. *Mol Biol Evol.* **35**(3), 773–777 (2018).

3. Stamatakis, A. RAxML version 8: A tool for phylogenetic analysis and post-analysis of large phylogenies. *Bioinformatics*. **30**(9), 1312–3 (2014).

4. Rambaut, A., Lam, T.T., Carvalho, L.M. & Pybus, O.G.. Exploring the temporal structure of heterochronous sequences using TempEst (formerly Path-O-Gen). *Virus Evol*. **2**(1), 10.1093/ve/vew007 (2016).

5. Suchard, M.A. *et al*. Bayesian phylogenetic and phylodynamic data integration using BEAST 1.10. *Virus Evol*. **4**(1), 10.1093/ve/vey016 (2018).

6. Minin, V.N., Bloomquist, E.W. & Suchard, M.A. Smooth skyride through a rough skyline: Bayesian coalescent-based inference of population dynamics. *Mol Biol Evol.* **25**(7),1459–1471 (2008).

7. Rambaut, A., Drummond, A.J., Xie, D., Baele, G. & Suchard, M.A. Posterior summarization in Bayesian phylogenetics using Tracer 1.7. *Syst Biol.* **67**(5), 901-904 (2018).

8. Lemey, P., Rambaut, A., Drummond, A.J. & Suchard, M.A. Bayesian phylogeography finds its roots. *PLoS Comput Biol*. **5**(9), 10.1371/journal.pcbi.1000520 (2009).

9. Oster, A.M. *et al*. Identifying clusters of recent and rapid HIV transmission through analysis of molecular surveillance data. *J Acquir Immune Def Syndr*. **79**(5), 543–550 (2018).

10. Ragonnet-Cronin, M., Hodcroft, E.B. & Wertheim, J.O. Understanding disclosed and cryptic HIV transmission risk via genetic analysis: What are we missing and when does it matter? *Curr Opin HIV AIDS.* **14**(3), 205–212 (2019).

11. Bahl, J. *et al*. Influenza A virus migration and persistence in North American wild birds. *PLoS Pathog*. **9**(8), 10.1371/journal.ppat.1003570 (2013).

12. Edwards, C.J. *et al*. Ancient hybridization and an Irish origin for the modern polar bear matriline. *Curr Biol.* **21**(15), 1251–1258 (2011).

13. Bielejec, F., Baele, G., Vrancken, B., Suchard, M.A., Rambaut, A. & Lemey, P. SpreaD3: Interactive visualization of spatiotemporal history and trait evolutionary processes. *Mol Biol Evol.* **33**(8), 2167-2169 (2016).
